# Metagenome Analysis of Intestinal Bacteria in Healthy People, Patients With Inflammatory Bowel Disease and Colorectal Cancer

**DOI:** 10.3389/fcimb.2021.599734

**Published:** 2021-02-26

**Authors:** Yongshun Ma, Yao Zhang, Houxiang Jiang, Shixin Xiang, Yueshui Zhao, Mintao Xiao, Fukuan Du, Huijiao Ji, Parham Jabbarzadeh Kaboli, Xu Wu, Mingxing Li, Qinglian Wen, Jing Shen, Zhongming Yang, Jing Li, Zhangang Xiao

**Affiliations:** ^1^Laboratory of Molecular Pharmacology, Department of Pharmacology, School of Pharmacy, Southwest Medical University, Luzhou, China; ^2^South Sichuan Institute of Translational Medicine, Luzhou, China; ^3^Department of Gastrointestinal Surgery, The First Affiliated Hospital of Wannan Medical College (Yijishan Hospital of Wannan Medical College), Wuhu, China; ^4^Department of Oncology, Affiliated Hospital of Southwest Medical University, Luzhou, China; ^5^Department of Oncology and Hematology, Hospital (T.C.M) Affiliated to Southwest Medical University, Luzhou, China; ^6^Department of Pharmacy, The Affiliated Hospital of Southwest Medical University, Luzhou, China

**Keywords:** inflammatory bowel disease, colorectal cancer, intestinal bacteria, taxonomic biomarkers, metagenomics, fecal microbiota

## Abstract

**Objectives:**

Several reports suggesting that the intestinal microbiome plays a key role in the development of inflammatory bowel disease (IBD) or colorectal cancer (CRC), but the changes of intestinal bacteria in healthy people, patients with IBD and CRC are not fully explained. The study aimed to investigate changes of intestinal bacteria in healthy subjects, patients with IBD, and patients with CRC.

**Materials:**

We collected data from the European Nucleotide Archive on healthy people and patients with colorectal cancer with the study accession number PRJEB6070, PRJEB7774, PRJEB27928, PRJEB12449, and PRJEB10878, collected IBD patient data from the Integrated Human Microbiome Project from the Human Microbiome Project Data Portal. We performed metagenome-wide association studies on the fecal samples from 290 healthy subjects, 512 IBD patients, and 285 CRC patients. We used the metagenomics dataset to study bacterial community structure, relative abundance, functional prediction, differentially abundant bacteria, and co-occurrence networks.

**Results:**

The bacterial community structure in both IBD and CRC was significantly different from healthy subjects. Our results showed that IBD patients had low intestinal bacterial diversity and CRC patients had high intestinal bacterial diversity compared to healthy subjects. At the phylum level, the relative abundance of Firmicutes in IBD decreased significantly, while the relative abundance of Bacteroidetes increased significantly. At the genus level, the relative abundance of *Bacteroides* in IBD was higher than in healthy people and CRC. Compared with healthy people and CRC, the main difference of intestinal bacteria in IBD patients was Bacteroidetes, and compared with healthy people and IBD, the main difference of intestinal bacteria in CRC patients was in Fusobacteria, Verrucomicrobia, and Proteobacteria. The main differences in the functional composition of intestinal bacteria in healthy people, IBD and CRC patients were L-homoserine and L-methionine biosynthesis, 5-aminoimidazole ribonucleotide biosynthesis II, L-methionine biosynthesis I, and superpathway of L-lysine, L-threonine, and L-methionine biosynthesis I. The results of stratified showed that the abundance of Firmicutes, Bacteroidetes, and Actinobacteria involved in metabolic pathways has significantly changed. Besides, the association network of intestinal bacteria in healthy people, IBD, and CRC patients has also changed.

**Conclusions:**

In conclusion, compared with healthy people, the taxonomic and functional composition of intestinal bacteria in IBD and CRC patients was significantly changed.

## Introduction

The incidence and mortality rate of IBD and CRC are very high and increase year by year (Ferlay et al., 2015; Dahlhamer et al., 2016). It is noteworthy that the human microbiome is becoming an area of increasing concern, and it is closely related to human status. There is growing evidence that gut microbes play an important role in IBD and CRC (Ni et al., 2017; Kwong et al., 2018). It is well-known that an altered gut microbiota composition is associated with IBD (Manichanh et al., 2012). Besides, patients with IBD are at increased risk of developing CRC in later life, and aberrant immune responses to penetrating commensal microbes may play key roles in promoting disease progression, but the reasons for this are not fully explained (Gillen et al., 1994; Munkholm, 2003; Rutter et al., 2004; Yu, 2018; Clarke and Feuerstein, 2019). Taxonomic and functional changes to the composition of the intestinal bacteria have been implicated in multiple human diseases, including IBD and CRC (Hall et al., 2017; Yu, 2018; Jackson and Theiss, 2020). Studies have shown that intestinal microorganisms may promote the development of cancer through metabolites (Louis et al., 2014). Previous studies have linked the development of CRC to the presence of *Bacteroides fragilis* in the intestinal microbiome (Toprak et al., 2006; Kwong et al., 2018; Zamani et al., 2019). Intestinal microbiota can affect the occurrence of colorectal cancer in various ways by promoting sustained inflammation and weakening host immunity (Park et al., 2018). Therefore, it is worthwhile to study the changes in intestinal bacteria from healthy people to IBD and CRC, which will contribute to further understanding of the pathogenesis of intestinal bacteria in diseases.

In the past, 16S rRNA gene sequencing was used to characterize the microbial community (Ahn et al., 2013; Wu et al., 2013; Mira-Pascual et al., 2015). Currently, the use of metagenomics for whole-genome shotgun sequencing is becoming more and more popular (Zeller et al., 2014; Feng et al., 2015; Yu et al., 2017). Analysis of the metagenomics dataset can reveal not only bacterial community structure but also the functions of microbial communities, and microbial high-throughput sequencing technology has made important contributions in revealing bacterial and human diseases (Qin et al., 2010; Maccaferri et al., 2011; Human Microbiome Project, 2012).

Here, we conducted the following studies: (i) to study the taxonomic and functional changes to the composition of intestinal bacteria in healthy people, patients with IBD and CRC; (ii) to study the changes of relative abundance of intestinal bacteria in healthy people, patients with IBD and CRC; (iii) to identify the species with the greatest difference; (iv) to investigate changes in bacterial interactions between healthy, IBD and CRC patients.

## Materials and Methods

### Study Subjects and Sample Collection

We downloaded a gut metagenomics dataset from the European Nucleotide Archive, which included 290 healthy people and 285 CRC patients, and 512 patients with IBD from the IBDMDB website (https://ibdmdb.org). The project PRJEB6070 included 61 healthy people and 53 CRC, the project PRJEB7774 included 63 healthy people and 46 CRC, the project PRJEB27928 included 60 healthy people and 60 CRC, the project PRJEB12449 included 52 healthy people and 52 CRC, the project PRJEB10878 included 74 healthy people and 54 CRC (**Table S1**). The metagenomics data of adult samples from this study were collected before treatment, thus excluding cancer therapy as a potential confounding effect.

### Analysis of Whole Metagenome Sequencing Data

We analyzed the collected metagenomics dataset using the same settings. (i) Raw reads were trimmed for quality using trimmomatic (Bolger et al., 2014) and retained high-quality sequences, then human host reads were subtracted by mapping the reads with the human reference genome (hg19) using Bowtie2 (Langmead and Salzberg, 2012). (ii) Fastq files were assessed for quality control using the FASTQC application. (iii) Taxonomically profiled using MetaPhlAn2 (Truong et al., 2015). Functional profiling was performed using HUMAnN2 (Abubucker et al., 2012), mapping to the UniRef90 database. (iv) Linear discriminant analysis effect size (LEFSe) (Segata et al., 2011) was used to identify differentially abundant bacteria species between two or more groups, and linear discriminant analysis (LDA) score was obtained. (v) STAMP (Parks et al., 2014) was used to identify differentially metabolic pathways. (vi) Correlation coefficients were determined using Spearman’s rank correlation, analyses were undertaken using the psych R package.

### Statistical Analysis

We estimated false discovery rates using the Storey method to adjust for multiple comparisons, to compare differences between groups. Spearman correlation coefficient (>0.4 or <−0.4) was used to construct the co-occurrence network, and Gephi (version 0.9.2) was used for visualization. Other statistical analyses were conducted using R (version 3.5.2). For the significantly different species wad used the nonparametric factorial Kruskl-wallis test, P < 0.05 was considered as a significant difference.

## Results

### Intestinal Bacterial Dysbiosis in IBD and CRC

**Figure 1:**
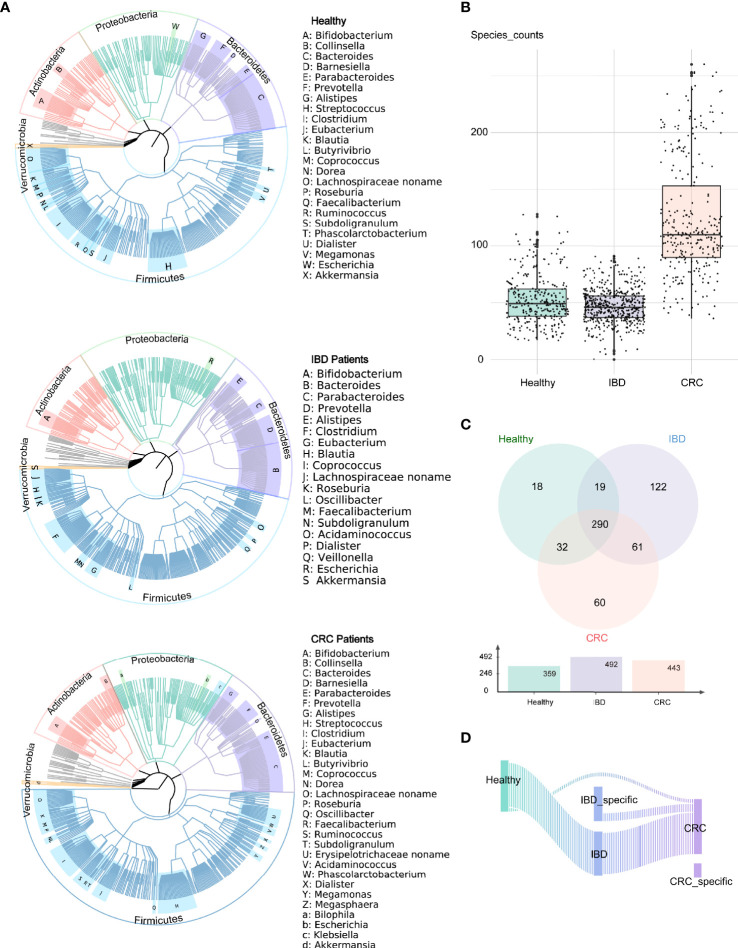
Altered bacterial microbiota biodiversity and composition in IBD and CRC. **(A)** The composition of intestinal bacteria in the healthy group, IBD group, and CRC group was displayed by three phylogenetic trees. Different colors represent different phyla level classification, red for Actinobacteria, light green for Proteobacteria, pale purple for Bacteroidetes, azure for Firmicutes, orange for Verrucomicrobia, and gray for other phyla with low abundance. The letters indicate the genus with higher abundance. **(B)** The box plot showed the differences in the number of bacterial at the species level among the three groups. **(C)** The Venn diagram showed the common species and unique bacterial species of the three groups. **(D)** The Sankey chart showed the change of bacteria from healthy groups to IBD to CRC.

We analyzed the bacterial fraction of the microbiota using metagenome sequencing data and found that the taxonomic composition of the gut bacteria of IBD and CRC has changed significantly. At the phylum level, the intestinal bacteria of healthy people, IBD and CRC patients was dominated by four phyla: Firmicutes, Bacteroidetes, Proteobacteria, Actinobacteria, among which, Firmicutes and Bacteroidetes are the most abundant bacteria in the gut microbiota (**Figure 1A**). At the species level, compared with CRC patients and healthy controls, the species counts of intestinal bacteria in patients with IBD decreased. Additionally, the number of species in CRC had increased in comparison to healthy controls and IBD (**Figure 1B** and **Table S2**). There were 290 common bacterial species in healthy people, IBD, and CRC (**Figure 1C**). IBD and CRC showed the different bacterial species from healthy people (**Figure 1D**).

### Changes in the Abundance of Intestinal Bacteria in Patients With IBD and CRC

**Figure 2:**
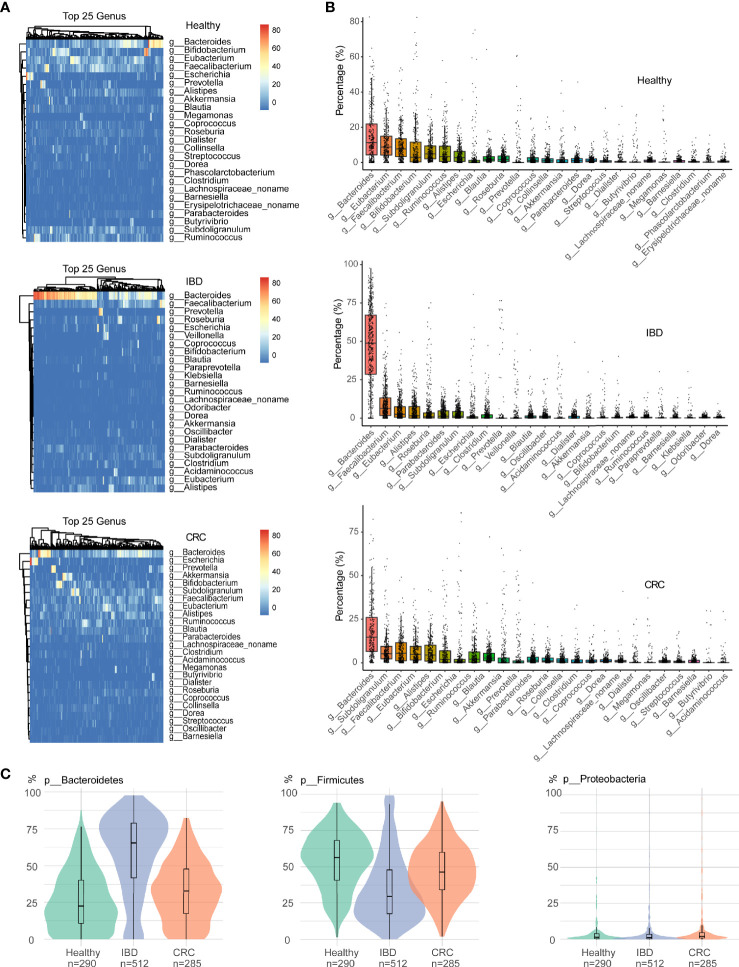
The difference in intestinal bacteria at the phylum and genus level among samples of different states. **(A)** The relative abundance of bacterial genera within the top 25 in healthy people, IBD, and CRC. **(B)** The boxplots showed the relative abundance of the top 25 genera in each group, sort the top 25 genera based on their mean values. **(C)** Bacterial relative abundance in the phylum level, including Firmicutes, Bacteroidetes, and Proteobacteria phylum. Green represents healthy controls, Purple represents IBD, and red represents CRC.

As shown in **Figure 2A**, the relative abundance of bacteria in the genus *Bacteroides* was the highest in healthy people, IBD, and CRC. At the genus level, the relative abundance of *Bacteroides*, *Eubacterium*, *Faecalibacaterium*, and *Bifidobacterium* was high in healthy people. The relative abundance of *Bacteroides*, *Faecalibacaterium*, *Eubacterium*, and *Alistipes* was high in IBD, and the relative abundance of *Bacteroides*, *Subdoligranulum*, *Faecalibacaterium*, and *Eubacterium* was high in CRC. More specifically, we found that *Bacteroides* abundance was significantly increased in IBD patients (**Figure 2B** and **Table S3**). At the species level, the top 25 species based on relative abundances were shown in the heatmap (**Figure S1A**). The relative abundance of *Faecalibacaterium_prausnitzii*, *Eubacterium_rectale*, *Ruminococcus_bromii*, and *Bifidobacterium_adolescentis* was high in healthy people. The relative abundance of *Faecalibacterium_prausnitzii*, *Bacteroides_uniformis*, *Bacteroides_vulgatus*, and *Bacteroides_stercoris* was high in IBD. The relative abundance of *Faecalibacterium_prausnitzii*, *Akkermansia_muciniphila*, *Eubacterium_rectale*, and *Ruminococcus_bromii* was high in CRC (**Figure S1B** and **Table S4**). We focused on the relative abundance of Proteobacteria, Firmicutes, and Bacteroidetes phyla. Compared with the healthy control group, the relative abundance of phylum Firmicutes decreased and phylum Bacteroidetes increased in IBD patients. The relative abundance of the phylum Firmicutes in CRC patients significantly increased compared with IBD, while the relative abundance of Bacteroidetes was decreased (**Figure 2C**).

### Bacterial Species With Significant Differences in Healthy People, Patients With IBD and CRC

**Figure 3:**
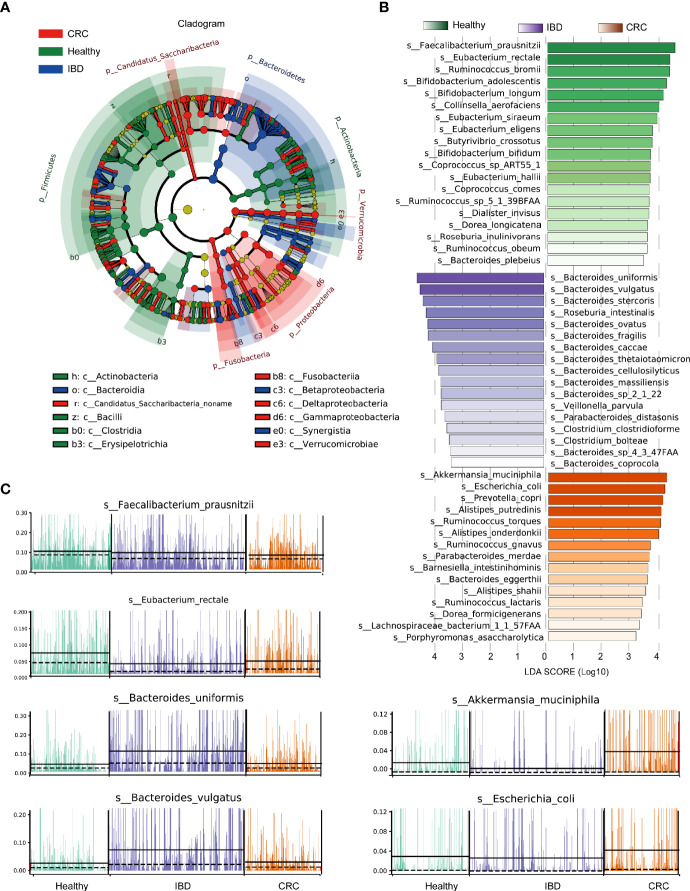
LEfSe analysis of the relative abundance of intestinal bacteria in healthy people, IBD, and CRC. **(A)** The node size represents the difference in relative abundance. Yellow nodes indicate bacteria with no significant differences in relative abundance. LEfSe cladogram in red for the taxa enriched in the CRC group, green for the taxa enriched in the healthy group, and blue for the taxa enriched in the IBD group. The meaning of shading color is the same as the node color. **(B)** The histogram showed all the different bacterial species and LDA scores, bacterial species with LDA score >2.0 and P < 0.05 were considered to be significantly discriminant. **(C)** The relative abundance of the top six bacterial species, each bar represents a patient sample.

The phylogenetic tree showed all the bacterial species that were significantly different in healthy people, patients with IBD, and CRC. The differential bacterial of the healthy group were mainly in phylum Firmicutes, Actinobacteria, the differential bacterial of the IBD group were mainly in phylum Bacteroidetes, and the differential bacterial of the CRC group were mainly in phylum Fusobacteria, Proteobacteria, and Verrucomicrobia (**Figure 3A**). LEfSe analysis showed that the healthy people were characterized by a higher abundance of *Faecalibacterium_prausnitzii*, *Eubacterium_rectale*, *Ruminococcus_bromii*, *Bifidobacterium_adolescentis*, *Bifidobacterium_longum*, *Collinsella_aerofaciens*. The IBD patients primarily showed higher enrichment with *Bacteroides_uniformis*, *Bacteroides_vulgatus*, *Bacteroides_stercoris*, *Roseburia_intestinalis*, *Bacteroides_ovatus*, *Bacteroides_fragilis*, *Bacteroides_caccae*. The CRC patients primarily showed higher enrichment with *Akkermansia_muciniphila*, *Escherichia_coli, Prevotella_copri*, *Alistipes_putredinis*, *Ruminococcus_torques* (LDA score >4.0 with *P* < 0.05) (**Figure 3B** and **Table S5**). A stacked barplot showed the relative abundance of 18 bacterial species with the most significant differences in healthy, IBD, and CRC (**Figure S2**). The relative abundance of *Faecalibacterium_prausnitzii*, *Eubacterium_rectale*, *Bacteroides_uniformis*, *Bacteroides_vulgatus*, *Akkermansia_muciniphila*, and *Escherichia_coli* in each sample was shown in **Figure 3C**.

### Functional Changes in the Composition of the Intestinal Bacterial in Patients With IBD and CRC and Healthy People

**Figure 4:**
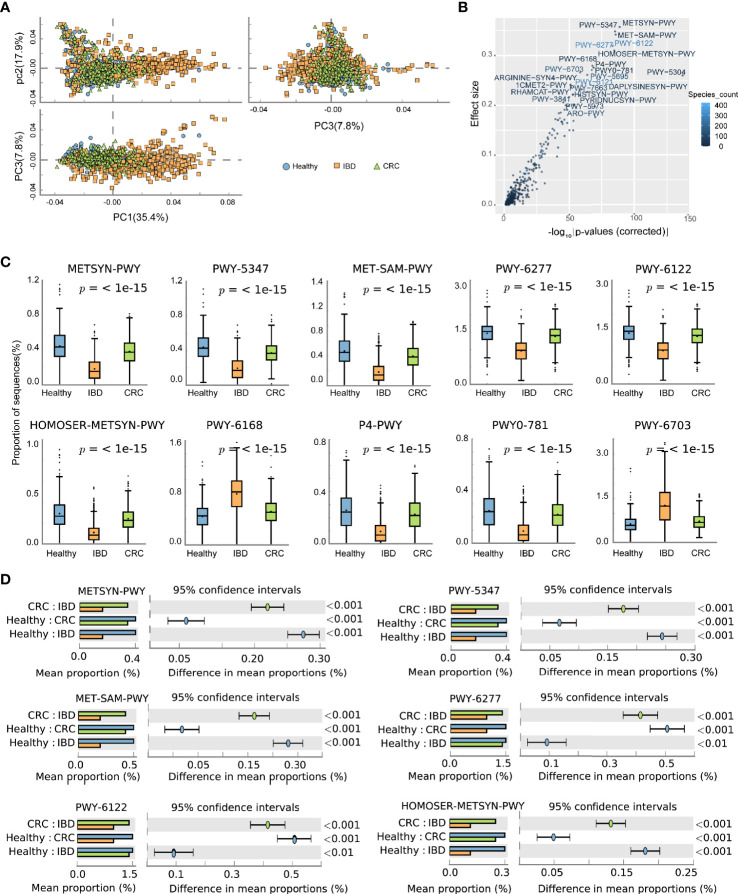
The effect of the altered intestinal bacteria on predicted functional metabolic pathways. **(A)** PCA analysis of intestinal bacterial metabolic pathway, the blue color represents the healthy, yellow represents the IBD, green represents the CRC. **(B)** The scatter plot showed the functional differences between the three groups. The x-coordinate represents the corrected -log10(P-values), the y-coordinate represents the effect size, and the color depth represents the number of species participating in the pathway. **(C)** The boxplots showed the changes in the relative abundance of bacteria involved in the 10 differential pathways. The lower and upper hinges of boxplots presented in the Figures correspond to the 25th and 75th percentiles, respectively. The midline is the median. Data beyond the end of the whiskers are plotted individually. **(D)** The bar chart shows the functional differences between groups.

The predicted functional potential of the gut bacteria was identified using HUMANn2. Principal component analysis (PCA) revealed differences in the functional composition of intestinal bacteria among the healthy people, IBD, and CRC, the variances accounted by principal component 1 and principal component 2 were 35.4 and 17.9%, respectively (**Figure 4A**). The scatter plot shows all the differential metabolic pathways between the healthy group, IBD group, and CRC group. Pathways with significant differences included METSYN-PWY (L-homoserine and L-methionine biosynthesis), PWY-5347 (superpathway of L-methionine biosynthesis), MET-SAM-PWY (superpathway of S-adenosyl-L-methionine biosynthesis), PWY-6122 (5-aminoimidazole ribonucleotide biosynthesis II), PWY-6277 (superpathway of 5-aminoimidazole ribonucleotide biosynthesis), HOMOSER-METSYN-PWY (L-methionine biosynthesis I), PWY-6168 [flavin biosynthesis III (fungi)], P4-PWY (superpathway of L-lysine, L-threonine and L-methionine biosynthesis I), PWY0-781 (aspartate superpathway), and PWY-6703 (preQ0 biosynthesis) in MetaCyc (**Figure 4B** and **Table S6**). The boxplots showed the changes in the abundance of bacteria that participate in important pathways. Compared with the healthy group, the relative abundance of bacteria involved in METSYN-PWY, PWY-5347, MET-SAM-PWY, PWY-6277, PWY-6122, HOMOSER-METSYN-PWY, P4-PWY, and PWY0-781 pathways in the IBD and the CRC group decreased. Compared with the healthy group, the relative abundance of bacteria involved in PWY-6168 and PWY-6703 pathways increased. In general, the relative abundance of bacteria involved in the pathway changed significantly in the IBD group (**Figure 4C**). We compared the differences of bacteria that participate in six important differential pathways, including healthy *versus* IBD, IBD *versus* CRC, and healthy *versus* CRC (**Figure 4D**). The main differential metabolic pathways between healthy people and IBD included PWY-6121 (5-aminoimidazole ribonucleotide biosynthesis I), PWY-6122 (5-aminoimidazole ribonucleotide biosynthesis II), PWY-6277 (superpathway of 5-aminoimidazole ribonucleotide biosynthesis), PWY-6703 (preQ0 biosynthesis). The main differential metabolic pathways between IBD and CRC included PWY-6703 (preQ0 biosynthesis), PWY-6700 (queuosine biosynthesis), PWY-2942 (L-lysine biosynthesis III) (**Figure S3**).

### Stratified Analyses of Metabolic Pathways by Bacterial Species

**Figure 5:**
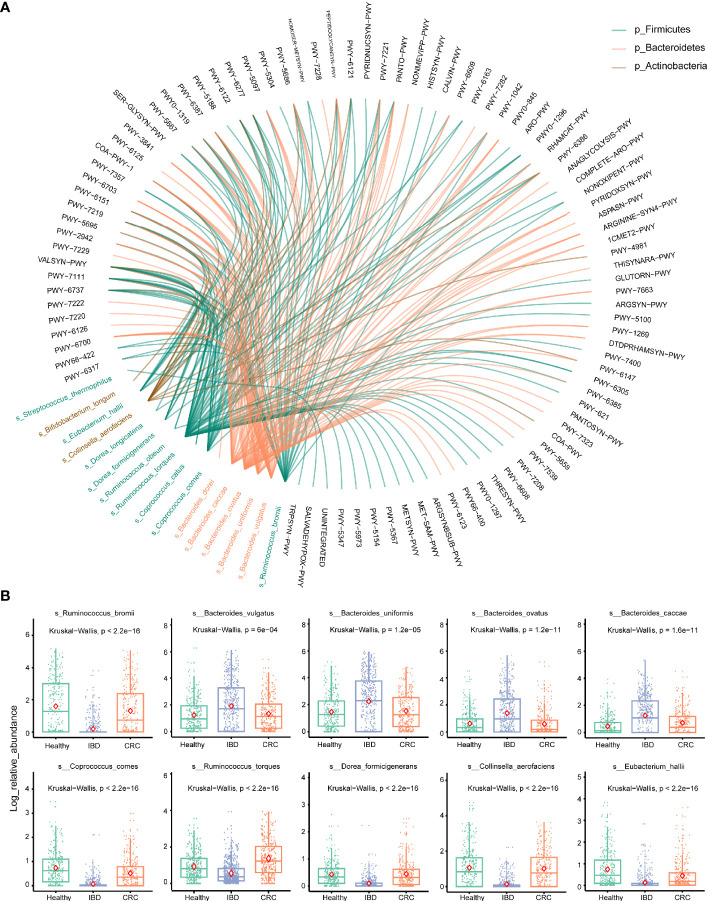
Stratified analyses of metabolic pathways by bacterial species. **(A)** The circle shows the pathways in which 16 differential bacteria species are involved. The black font represents the metabolic pathways. Red represents the bacterium that belongs to the phylum Bacteroidetes, green represents the bacterium that belongs to the phylum Firmicutes, brown represents the bacterium that belongs to the phylum Actinobacteria. **(B)** Log abundance for 10 major bacteria species compared between Healthy, IBD, and CRC cases. P values were determined by Kruskal–Wallis tests. The midline is the median, and the red diamond shape represents the mean values.

To account for differences of bacterial species involved in metabolic pathways, we stratified analyses by bacterial species. We used the circle diagram to show the connections between bacterial species and the pathways (Effect size >0.6 with p-values [corrected] <0.05) (**Table S7**). The bacterial species participate in many different pathways, including METSYN-PWY (L-methioninebiosynthesisI), PWY-5347 (superpathway of L-methionine biosynthesis), MET-SAM-PWY (superpathway of S-adenosyl-L-methionine biosynthesis), PWY-6122 (5-aminoimidazole ribonucleotide biosynthesis II), PWY-6277 (superpathway of 5-aminoimidazole ribonucleotide biosynthesis), HOMOSER-METSYN-PWY (L-methionine biosynthesis I) (**Figure 5A**). The differences in the relative abundance of 10 major bacteria were shown in **Figure 5B**.

### The Bacterial Co-occurrence Network Was Different in Healthy People, IBD, and CRC

**Figure 6:**
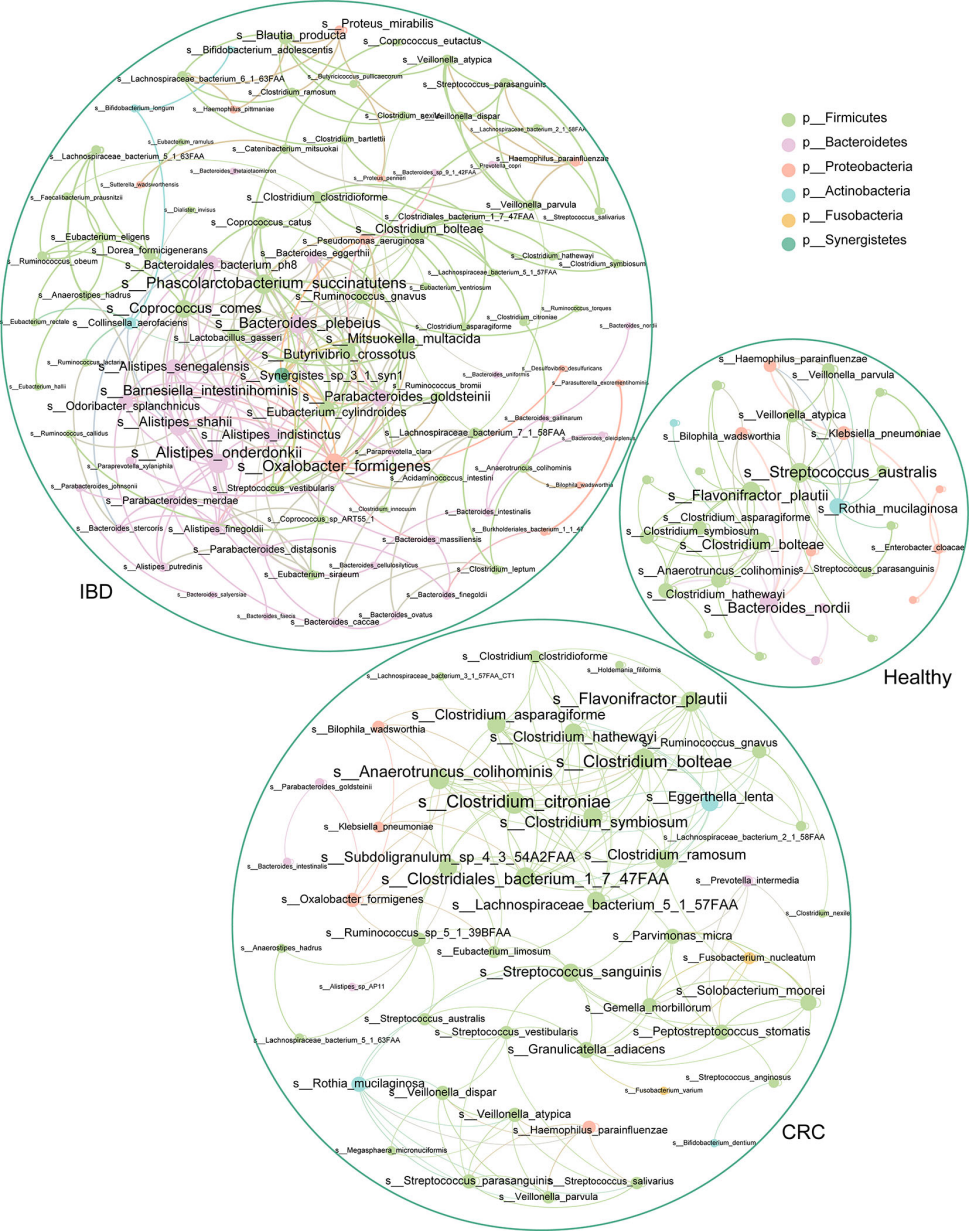
The association network of intestinal bacteria in different states. Each circle (node) represents a bacterial species, its color represents the bacterial phylum it belongs to and its size represents the number of direct edges that it has. Only significant correlations (−0.4<r<0.4 and p < 0.05) are displayed.

To understand the interaction between intestinal bacteria at the species level in healthy people, IBD, and CRC patients, we constructed three co-occurrence networks. Spearman correlation coefficient was used to evaluate the relationship between the relative abundance of each bacterial species in different samples. The degree centrality of *Flavonifractor_plautii*, *Bacteroides_nordii*, *Streptococcus_australis* in the healthy group was high. Analysis of the IBD group shows that *Oxalobacter_formigenes*, *Alistipes_onderdonkii*, *Bacteroides_plebeius*, *Coprococcus_comes*, and *Phascolarctobacterium_succinatutens* had the high degree centrality. In CRC groups, we found that *Clostridium_citroniae*, *Clostridiales_bacterium_1_7_47FAA*, *Clostridium_bolteae*, *Anaerotruncus_colihominis*, and *Flavonifractor_plautii* had a high degree of centrality (**Figure 6**).

## Discussion

The human colon contains a large number of bacteria, which play an important role in the regulation of immune function (Hooper et al., 2012; Kabat et al., 2014). The microbial community structure is different, which may be related to the environment, diet, and other factors (David et al., 2014; Gaulke and Sharpton, 2018). A high intake of red meat has been reported to contribute to bacterial growth, leading to a hostile intestinal environment, and there is a metabolic link between cancer-associated gut microbes and fat- and meat-rich diet (Feng et al., 2015; Tilg et al., 2018; Wirbel et al., 2019). More and more researchers have realized that intestinal bacterial dysbiosis is a major cause of multiple diseases, including IBD and CRC. Next-generation sequencing technologies have identified alteration in the composition and function of the intestinal microbiome in IBD (Matsuoka and Kanai, 2015; Nishida et al., 2018). However, there is no unified study on the changes in intestinal bacteria from normal people to IBD and CRC, and we conducted a meta-analysis here. We found the taxonomic and functional composition of intestinal bacteria in IBD and CRC was greatly changed, and the change of their relative abundance mainly of Firmicutes, Actinobacteria, Bacteroidetes, Fusobacteria, Verrucomicrobia, and Proteobacteria phylum (**Figure 3A**).

At the phylum level, the relative abundance of Firmicutes decreased and Bacteroidetes increased in IBD patients (**Figure 2C**). This finding is consistent with the results reported previously (Gophna et al., 2006; Manichanh et al., 2006; Frank et al., 2007). The relative abundance of *Bacteroides* was the highest in healthy people, IBD, and CRC compared with other bacteria genera (**Figures 2A, B**). At the species level, the number of species was higher in CRC patients than that in healthy and IBD (**Figure 1B**), which may be related to the increase of harmful bacteria in CRC (Wang et al., 2012). The difference in the functional composition of intestinal bacteria in healthy people, IBD, CRC were METSYN-PWY (L-homoserine and L-methionine biosynthesis), PWY-5347 (superpathway of L-methionine biosynthesis), MET-SAM-PWY (superpathway of S-adenosyl-L-methionine biosynthesis), PWY-6122 (5-aminoimidazole ribonucleotide biosynthesis II), PWY-6277 (superpathway of 5-aminoimidazole ribonucleotide biosynthesis), HOMOSER-METSYN-PWY (L-methionine biosynthesis I), P4-PWY (superpathway of L-lysine, L-threonine and L-methionine biosynthesis I), and PWY0-781 (aspartate superpathway) (**Figure 4B**). We found that many metabolic pathways changed in the IBD group, and the other study also reported that most metabolites were down-regulated in a mouse model (Lu et al., 2012). These results suggest that the utilization of host amino acids by intestinal bacteria is different in healthy people, IBD, and CRC. Besides, PWY-6737 (starch degradation V), PWY-6126 (superpathway of adenosine nucleotides *de novo* biosynthesis II), PWY-7229 (superpathway of adenosine nucleotides *de novo* biosynthesis I), PWY-6125 (superpathway of guanosine nucleotides *de novo* biosynthesis II), PWY-7219 (adenosine ribonucleotides *de novo* biosynthesis), and PWY-3841 (folate transformations II) pathways have also changed (**Figure 5A**), and the other study also reported that folate biosynthesis, starch degradation pathway, nucleotides metabolism, energy metabolism and intermediates of amino acids were affected (Kolho et al., 2017; Yusof et al., 2018; Thomas et al., 2019). Moreover, the change of PWY-6737 was mainly related to the change of Firmicutes (*Ruminococcus_bromii*; *Coprococcus_comes*; *Ruminococcus_torques*; *Dorea_formicigenerans*; *Eubacterium_hallii*). Compared with the healthy controls and CRC patients, the relative abundances of *Ruminococcus_bromii*, *Coprococcus_comes*, *Ruminococcus_torques*, *Dorea_formicigenerans*, and *Eubacterium_hallii* were down-regulated in IBD. The change of PWY-6126, PWY-7229, PWY-6125, PWY-7219, and PWY-3841 was mainly related to the change of Bacteroidetes (*Bacteroides_vulgatus*; *Bacteroides_ovatus*; *Bacteroides_uniformis*; *Bacteroides_caccae*). Compared with the healthy controls, the relative abundances of *Bacteroides_vulgatus*, *Bacteroides_ovatus*, *Bacteroides_uniformis*, *Bacteroides_caccae* were up-regulated in IBD (**Figure 5B**).

At the species level, the healthy people were characterized by a higher abundance of Faecalibacterium_prausnitzii, Eubacterium_rectale, Ruminococcus_bromii, Bifidobacterium_adolescentis, Bifidobacterium_longum, and Collinsella_aerofaciens. The IBD patients were characterized by a higher abundance of Bacteroides_uniformis, Bacteroides_vulgatus, Bacteroides_stercoris, Roseburia_intestinalis, Bacteroides_ovatus, Bacteroides_fragilis, and Bacteroides_caccae. The CRC patients were characterized by a higher abundance of Akkermansia_muciniphila, Escherichia_coli, Prevotella_copri, Alistipes_putredinis, and Ruminococcus_torques (**Figure 3B**). Bacteroides_fragilis is a significant source of chronic inflammation and has been implicated as a risk factor for colorectal cancer (Wu et al., 2009; Goodwin et al., 2011). Besides, Escherichia coli has been associated with IBD (Martinez-Medina and Garcia-Gil, 2014; Palmela et al., 2018; Mirsepasi-Lauridsen et al., 2019). Several reports in the literature highlighted that the amount of Faecalibacterium_prausnitzii negatively correlated with the IBD and CRC (Lopez-Siles et al., 2016; Quevrain et al., 2016; Zhou et al., 2018). Alistipes has been implicated in CRC (Parker et al., 2020). We expect that intestinal bacteria can be used for early diagnosis of IBD and CRC or can serve as prognostic markers. Moreover, the control of dysfunctional intestinal bacteria may become a target for future personalized medicine.

The microbial community in the human intestinal tract is a huge and complex system. In order to better study intestinal microbes, we need to do more work: (i) to investigate the effects of bacterial metabolites on the intestinal tract; (ii) to conduct animal experiments and clinical studies; (iii) to study the continuing dynamics changes of gut bacteria from healthy people to IBD to CRC; (v) to investigate the differences in intestinal bacteria between different states of patients. For example, the IBD subgroups (Crohn’s disease and ulcerative colitis), the activity, extent, history, treatment of the disease, and the characteristics of the patients (age, sex, diet) should be further studied. We believe that understanding the gut microbiota will provide safer and more effective treatments for microbial diseases.

## Data Availability Statement

The original contributions presented in the study are included in the article/**Supplementary Material**. Further inquiries can be directed to the corresponding authors.

## Author Contributions

ZX, JL, and ZY supervised the project. YM, YaZ, and HXJ designed experiments and analyzed data. SX, YuZ, MX, FD, HJJ, and PK collected the data and wrote the manuscript. XW, ML, QW, and JS provided scientific expertise. All authors contributed to the article and approved the submitted version.

## Funding

This work was supported by National Natural Science Foundation of China (No.81672444, 81972643) and Sichuan Science and Technology Project (2018JY0079).

## Conflict of Interest

The authors declare that the research was conducted in the absence of any commercial or financial relationships that could be construed as a potential conflict of interest.

## Supplementary Material

Supplementary Table 1 Samples included in this study.

Supplementary Table 2 Species counts of healthy people, IBD, and CRC.

Supplementary Table 3 The top 25 genera with the highest relative abundance among samples.

Supplementary Table 4 The top 25 species with the highest relative abundance among samples.

Supplementary Table 5 The results of LDA analysis in healthy subjects, IBD, and CRC.

Supplementary Table 6 The differential pathways of healthy people, IBD, and CRC.

Supplementary Table 7 The results of stratified analyses of bacterial metabolic pathways.

Supplementary Figure 1 The top 25 species with the highest relative abundance in healthy people, IBD, and CRC.

Supplementary Figure 2 Stacked barplot of 18 most significant differences species among samples.

Supplementary Figure 3 The differential metabolic pathways between healthy and IBD, and between IBD and CRC.

## Abbreviations

IBD, inflammatory bowel disease; CRC, colorectal cancer; LDA, linear discriminant analysis; LEfSe, linear discriminant analysis effect size.
