# Corrigendum: Metagenome Analysis of Intestinal Bacteria in Healthy People, Patients With Inflammatory Bowel Disease and Colorectal Cancer

**DOI:** 10.3389/fcimb.2021.765843

**Published:** 2021-09-27

**Authors:** Yongshun Ma, Yao Zhang, Houxiang Jiang, Shixin Xiang, Yueshui Zhao, Mintao Xiao, Fukuan Du, Huijiao Ji, Parham Jabbarzadeh Kaboli, Xu Wu, Mingxing Li, Qinglian Wen, Jing Shen, Zhongming Yang, Jing Li, Zhangang Xiao

**Affiliations:** ^1^ Laboratory of Molecular Pharmacology, Department of Pharmacology, School of Pharmacy, Southwest Medical University, Luzhou, China; ^2^ South Sichuan Institute of Translational Medicine, Luzhou, China; ^3^ Department of Gastrointestinal Surgery, The First Affiliated Hospital of Wannan Medical College (Yijishan Hospital of Wannan Medical College), Wuhu, China; ^4^ Department of Oncology, Affiliated Hospital of Southwest Medical University, Luzhou, China; ^5^ Department of Oncology and Hematology, Hospital (T.C.M) Affiliated to Southwest Medical University, Luzhou, China; ^6^ Department of Pharmacy, The Affiliated Hospital of Southwest Medical University, Luzhou, China

**Keywords:** inflammatory bowel disease, colorectal cancer, intestinal bacteria, taxonomic biomarkers, metagenomics, fecal microbiota

## Figure/Table Legend

In the original article, there was a mistake in the legend for **Figure 5** as published. There is a duplicate statement. The correct legend appears below.

The authors apologize for this error and state that this does not change the scientific conclusions of the article in any way. The original article has been updated.

## Correct legend

**Figure 5** Stratified analyses of metabolic pathways by bacterial species. **(A)** The circle shows the pathways in which 16 differential bacteria species are involved. The black font represents the metabolic pathways. Red represents the bacterium that belongs to the phylum Bacteroidetes, green represents the bacterium that belongs to the phylum Firmicutes, brown represents the bacterium that belongs to the phylum Actinobacteria. **(B)** Log abundance for 10 major bacteria species compared between Healthy, IBD, and CRC cases. P values were determined by Kruskal–Wallis tests. The midline is the median, and the red diamond shape represents the mean values.

## Incorrect Author Name

An author name was incorrectly spelled as Jianghou Xiang. The correct spelling is Houxiang Jiang.

The authors apologize for this error and state that this does not change the scientific conclusions of the article in any way. The original article has been updated.

An author name was incorrectly spelled as Zhongmin Yang. The correct spelling is Zhongming Yang.

The authors apologize for this error and state that this does not change the scientific conclusions of the article in any way. The original article has been updated.

## Publisher’s Note

All claims expressed in this article are solely those of the authors and do not necessarily represent those of their affiliated organizations, or those of the publisher, the editors and the reviewers. Any product that may be evaluated in this article, or claim that may be made by its manufacturer, is not guaranteed or endorsed by the publisher.

