# Supplementary figures and images for: Metagenome Analysis of Intestinal Bacteria in Healthy People, Patients With Inflammatory Bowel Disease and Colorectal Cancer
Source: Front Cell Infect Microbiol. 2021 Feb 26;11:599734. doi: 10.3389/fcimb.2021.599734 (PMC7962608; doi:10.3389/fcimb.2021.599734)

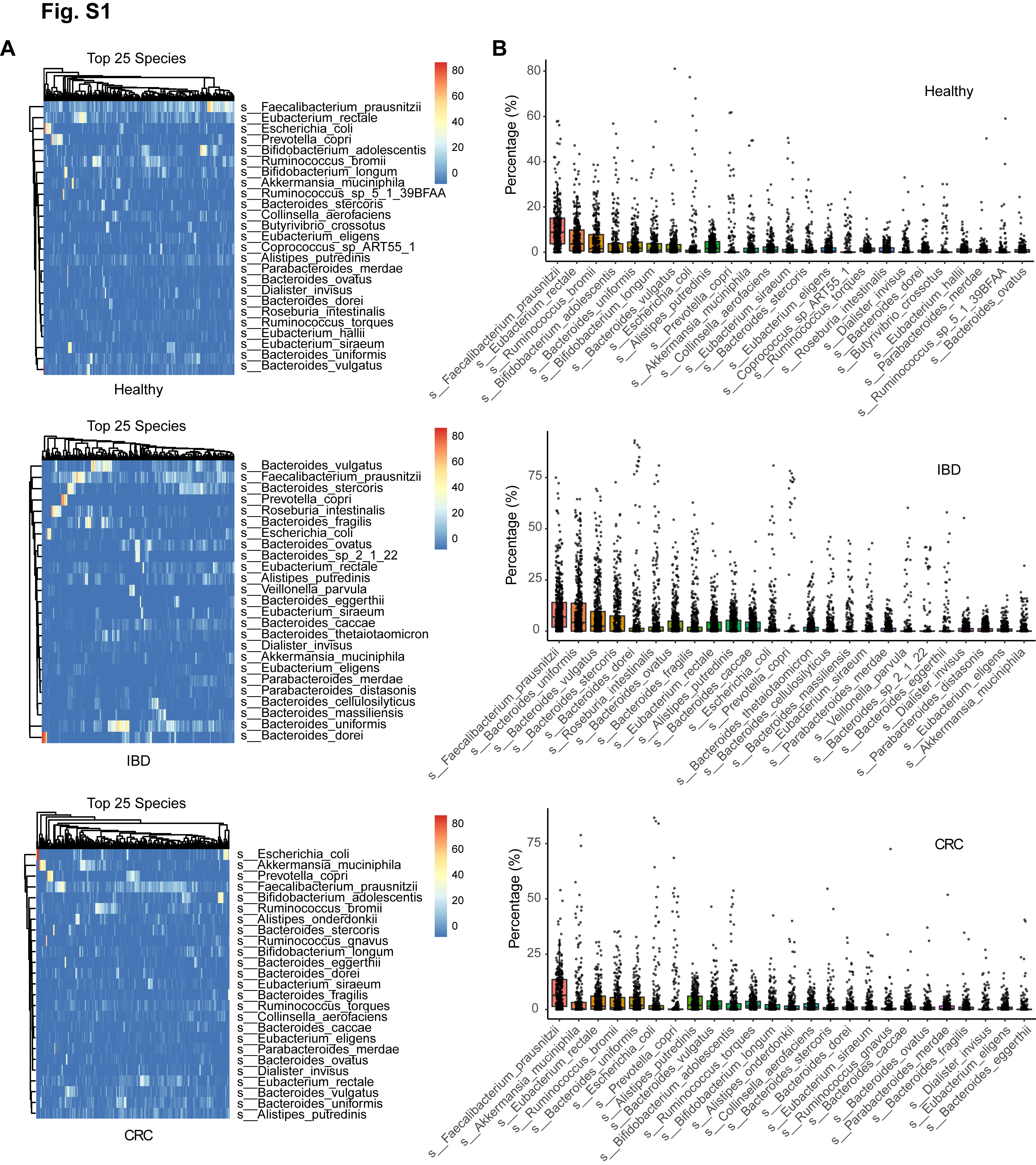

Supplement: Supplementary Figure 1 — The top 25 species with the highest relative abundance in healthy people, IBD, and CRC. [file Image_1.tif]

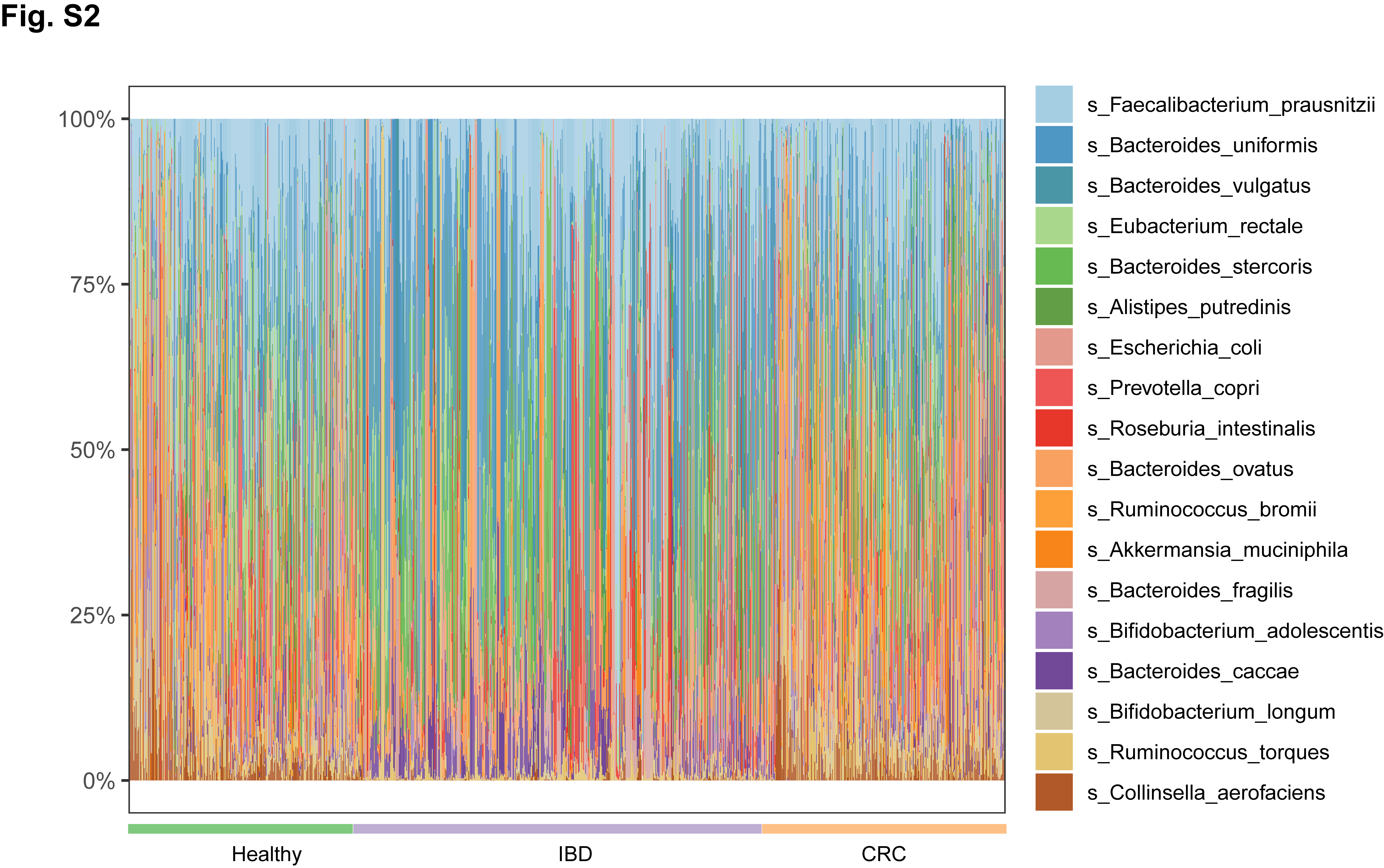

Supplement: Supplementary Figure 2 — Stacked barplot of 18 most significant differences species among samples. [file Image_2.tif]

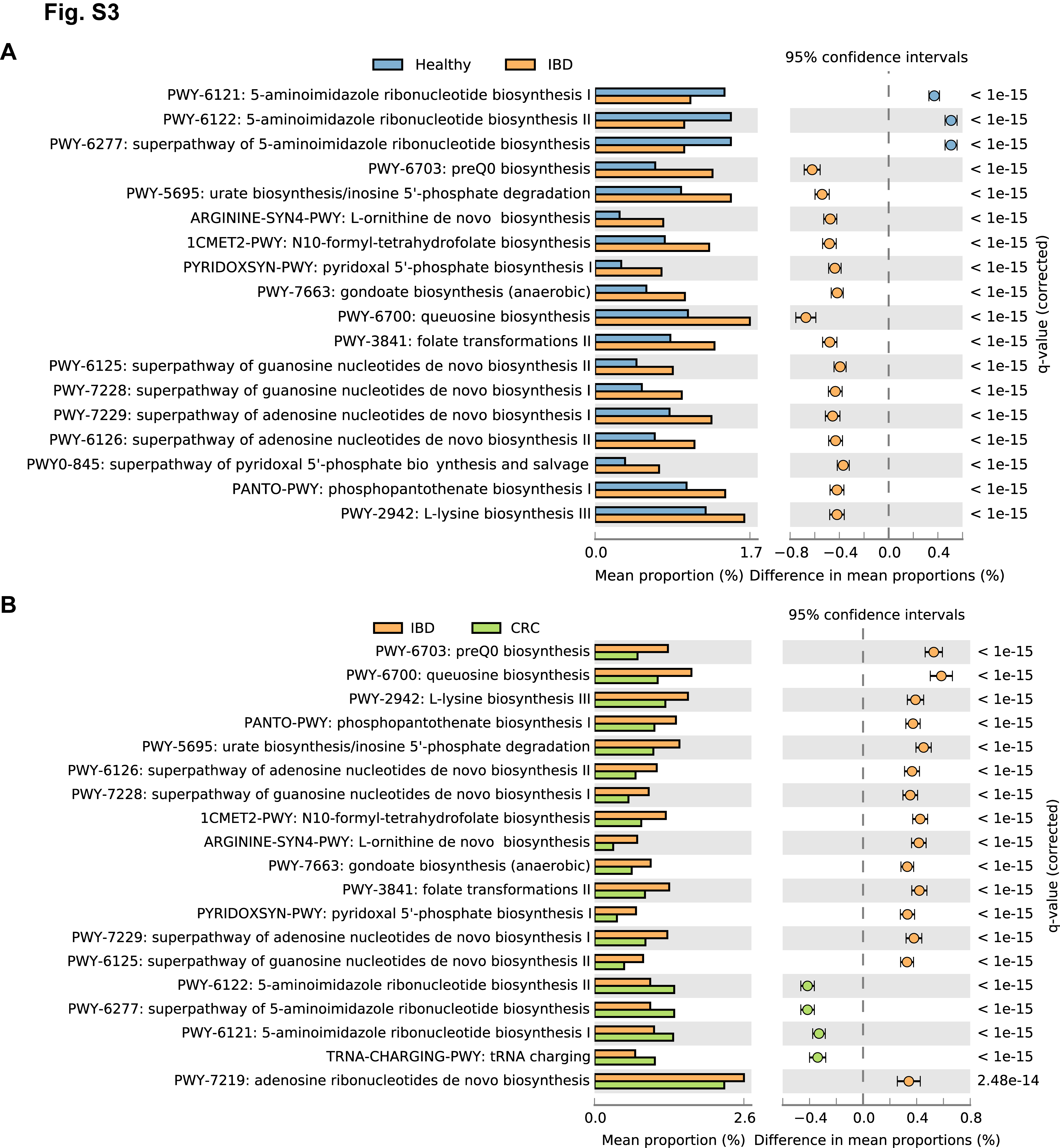

Supplement: Supplementary Figure 3 — The differential metabolic pathways between healthy and IBD, and between IBD and CRC. [file Image_3.tif]
